# Electrocardiographic left ventricular hypertrophy is not associated with increased in-hospital adverse events in patients with first Non-ST segment elevation myocardial infarction: A single center study

**DOI:** 10.22088/cjim.10.3.289

**Published:** 2019

**Authors:** Fatemeh Bakhtiari, Ghiti Davarmoin, Samad Ghaffari, Naser Aslanabadi, Ahmad Separham

**Affiliations:** 1 Cardiovascular Research Center, Cardiology Department, Tabriz University of Medical Science, Tabriz, Iran

**Keywords:** Non ST-segment elevation myocardial infarction (NSTEMI), Left ventricular hypertrophy (LVH), Electrocardiography (ECG), in-hospital mortality

## Abstract

**Background:**

There is conflicting data about prognostic implication of electrocardiographic (ECG) left ventricular hypertrophy (LVH) in patients with first non- ST-segment elevation myocardial infarction (NSTEMI). We aimed to examine the association of left ventricular hypertrophy (LVH) on admission electrocardiogram with adverse outcomes in patients with NSTEMI.

**Methods:**

In the present study, 460 patients (77.5% males with mean age of 65.44±13.15 years) with first NSTEMI were evaluated. ECG left ventricular hypertrophy (LVH) was diagnosed based on Sokolow-Lyon voltage criteria. Baseline laboratory and clinical results, angiographic data, as well as in- hospital adverse events were compared between the patients with and without LVH.

**Results:**

Electrocardiographic LVH was observed in 74 (16.1%) patients. Patients with LVH had higher admission systolic blood pressure (132.91±21.08 vs 125.80±21.78; P=0.01) and higher peak troponin (6.42±1.03 vs 4.41±0.28; P=0.004), but less likely to undergo coronary angiography (54.1% vs 66.8%; P=0.03) .Patients with electrocardiographic LVH had similar in-hospital mortality (5.4% vs 3.6%, P=0.5) and heart failure/ pulmonary edema (2.7% vs 2.07%, P=0.6) compared to patients without LVH.

**Conclusion:**

The present study showed that among the patients with first NSTEMI, electrocardiographic LVH was not associated with increased in-hospital adverse events.

Electrocardiographic evidence of left ventricular hypertrophy (LVH) is an independent and powerful determinant of cardiovascular death. This finding is often associated with a high probability of blood pressure-caused cardiovascular complications such as coronary artery disease, heart failure, stroke, and overall mortality (1-6). Left ventricular hypertrophy is caused by long-term and often untreated hypertension. By progressing LVH, the oxygen requirements of myocardium has increased, which can worsen supply-demand mismatch and potentially leads to acute coronary events (7). Therefore, diagnosis of patients with LVH is an important component of clinical risk reduction strategies in hypertensive patients. Numerous studies evaluated the relationship between electrocardiographic LVH and clinical outcomes in patients with ST-segment myocardial infarction (STEMI) or non-ST segment myocardial infarction.

Although many of these studies showed association between electrocardiographic LVH and cardiovascular outcomes like death and heart failure during hospital course and long-term follow-up (8-11), others showed that LVH in electrocardiogram had no prognostic implication (12-14). Given the contradiction in previous studies, we investigated the effect of electrocardiographic LVH on the in-hospital outcomes of patients with first NSTEMI.

## Methods

In the present study, all patients admitted with a diagnosis of non-ST segment elevation myocardial infarction between January 2015 and March 2017 in our tertiary center in northwest of Iran were enrolled. The diagnosis of NSTEMI was made using the third universal definition of myocardial infarction (15): typical anginal chest pain, elevated cardiac enzymes and ST-segment depression or T wave inversion. We excluded patients with a non-interpretable electrocardiogram, Left bundle branch block, acute ST-segment myocardial infarction, Paced rhythm, previous history of myocardial infarction, and previous history of any type of revascularization including coronary artery bypass grafting or percutaneous coronary intervention.

 All demographic and clinical findings, including age, gender, coronary risk factors, history of angina, history of medications, and hemodynamic status during initial presentation, including systolic blood pressure (SBP) and heart rate were recorded. Moreover, laboratory data and coronary angiography results as well as revascularization procedures were recorded. Major adverse cardiovascular events (MACE) were defined as cardiovascular mortality, reinfarction and heart failure. LVH on ECG was defined based on Sokolow and Lyon voltage criteria: S amplitude in lead V1 plus R amplitude in lead V5 or V6 ≥35 mm and/or R amplitude in lead V5 or V6 >26 mm (16). The present study complied with the Declaration of Helsinki and was approved by the institutional review board of our center and all patients gave written informed consent.

**Statistical analysis: **Categorical variables were described as frequency and percentage, and continuous variables, as mean± standard deviation. Chi-square test and Fisher's exact test were used to compare categorical variables between groups with and without electrocardiographic LVH, and independent t-test was used to compare the continuous variables. Furthermore, multivariate logistic tests were used to determine independent predictors of in-hospital complications as defined among the variables associated with p<0.05 in univariate analysis. In the present study, a p-value less than 0.05 was considered significant. All data were analyzed using SPSS 17 software. 

## Results

In the present research, 460 patients consisting of 306 (66.5%) males and 154 (33.5%) females with the mean age of 65.44±13.5 years were evaluated. Based on Sokolow and Lyon voltage criteria, LVH was observed in 74 (16.1%) patients. Basic and laboratory findings of patients with and without LVH are presented in table 1.

**Table 1:** Baseline characteristics of the study population

	**LVH** * **N=74**	**No LVH** **N=386**	**p-value**
Age (years)	67.52±14.87	65.08±12.82	0.1
Female, n (%)	18(24.3%)	136(35.2%)	0.06
Hypertension, n (%)	42(56.8%)	239(61.9%)	0.4
Diabetes mellitus, n (%)	34(45.9%)	132(34.2%)	0.05
Hyperlipidemia, n (%)	12(16.2%)	64(16.6%)	0.9
Previous angina, n (%)	23(31.08%)	112(29.01%)	0.7
Previous stroke, n (%)	2(2.7%)	14(3.6%)	0.6
Heart rate (beats/min)	80.47±16.06	84.56±19.2	0.08
Systolic blood pressure (mm Hg)	132.91±21.08	125.80±21.78	0.01
Killip class I, n (%)	61(82.4%)	305(79.01%)	0.6
LV ejection fraction (%)	43.1±3.8	45.1±3.6	0.3
More than Moderate Mitral regurgitation	24(31.5%)	123(31.8%)	0.9
Peak Creatine kinase-MB (U/L)	136.69±34.04	74.97±5.03	0.001
Peak Troponin I (ng/ml)	6.42±1.03	4.14±0.28	0.004
Creatinine (mg/dl)	1.19±0.64	1.35±1.25	0.3
Medication use before admission			
Aspirin, n (%)	26(35.1%)	131 (33.9%)	0.8
Beta-blocker, n (%)	40(54.1%)	171(44.3%)	0.1
Angiotensin-converting enzyme inhibitor, n (%)	8(10.8%)	23(5.9%)	0.1
Calcium antagonist, n (%)	8(10.8%)	24(6.2%)	0.2
Nitrate, n (%)	14(18.9%)	50(12.9%)	0.1

* LVH: Left ventricular hypertrophy

**Figure1:**
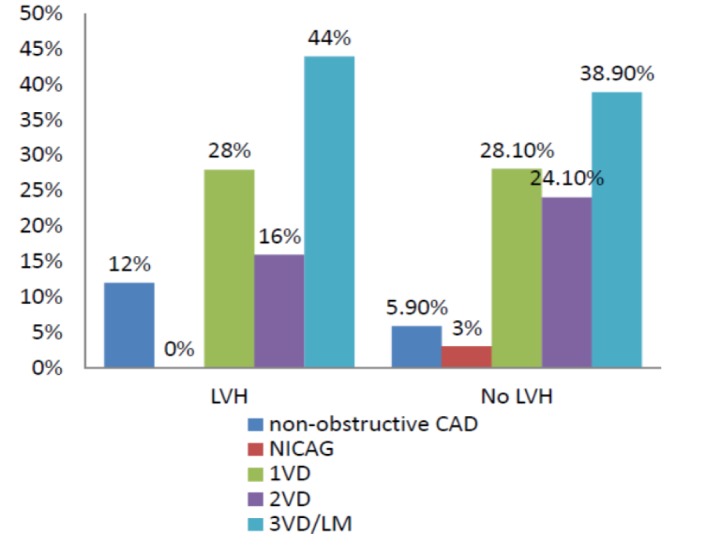
Coronary artery involvement in patients with and without LVH

 Patients with LVH presented with higher SBP and had higher levels of peak Troponin and CK-MB. Other characteristics and risk profile and medication history were similar between groups. Also, patients with LVH underwent fewer catheterizations than those without LVH (54.1% vs 66.8%; P=0.03). There was no difference regarding advanced coronary artery stenosis (LM/3VD) between patients with versus LVH (P0.26). (figure1)

NLCAG: NL coronary angiography, 1VD: Monovessel disease, 2VD: two-vessel disease, 3VD: three vessel disease, LM: left main coronary artery stenosis LVH: left ventricular Hypertrophy, CAD: coronary artery disease

**Figure 2:**
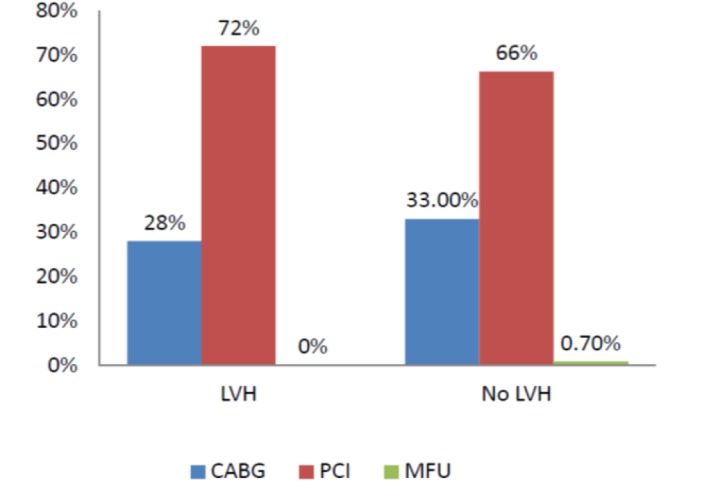
Revascularization procedure between patients with and without LVH

LVH: left ventricular hypertrophy; CABG: coronary artery bypass graft surgery; PCI: percutaneous coronary intervention; MFU: medical follow-up

Most of patients underwent coronary angioplasty as revascularization procedure. Percutaneous or surgical revascularization rate was not different between groups (p = 0.63) (Figure2). Patients with electrocardiographic LVH had similar in-hospital mortality (5.4% vs 3.6%, P=0.4), heart failure/ pulmonary edema (2.7% vs 2.07%, P=0.6) and Re-MI compared to patients without LVH (1.3% vs 0.25%, p=0.2) (table2).

**Table 2:** In-hospital major adverse cardiac events

	**LVH** * **N=74**	**No LVH** **N=386**	**p Value**
Mortality	4(5.4%)	14(3.6%)	0.4
Heart failure/pulmonary edema	2(2.7%)	8(2.07%)	0.6
Reinfarction	1(1.3%)	1(0.25%)	0.2

* LVH: Left ventricular hypertrophy

**Table3:** Univariate analysis for in-hospital major adverse cardiac events (MACE)

	**With MACE** * **N=30**	**Without MACE** **N=430**	**Pvalue**
Age(years)	68.64±9.91	65.26±13.29	0.2
Female, n (%)	14(46.6%)	140(32.5%)	0.1
Hypertension, n (%)	16(53.3%)	265(61.6%)	0.2
Diabetes mellitus, n (%)	8(26.6%)	158(36.7%)	0.9
Hyperlipidemia, n (%)	10(33.3%)	96(22.3%)	0.1
Previous angina, n (%)	18(60%)	372(86.5%)	0.6
Previous stroke, n (%)	2(6.6%)	14(3.2%)	0.1
Heart rate (beats/min)	105.4±4.1	99.2±16.7	0.04
Systolic blood pressure (mm Hg)	119.8±20.7	127.3±21.8	0.1
Creatinine (mg/dl)	1.84±0.09	1.35±0.9	0.003
Peak creatine kinase-MB (U/L)	103.4±5.2	87.9±26.1	0.001
Peak troponin I (ng/ml)	7.27±1.06	4.39±0.3	0.004
Electrocardiographic left ventricular hypertrophy (LVH), n (%)	4(13.3%)	70(16.2%)	0.8
LV ejection fraction (%)	38.4±9.3	41.2±10.1	0.1

* Major adverse cardiovascular events

Table 3 demonstrates basic and laboratory findings among patients with and without in-hospital MACE. Univariate predictors of in- hospital MACE were: higher levels of CK-MB, cardiac troponin, creatinine and tachycardia at admission. In multivariate logistic regression, none of the abovementioned factors could predict in-hospital MACE independently (table 4).

**Table 4:** Multivariate regression analysis of independent predictors of in-hospital MACE (major adverse cardiac events)

	**P value**	**Odds ratio**	**95% Confidence Interval**	
			**Lower**	**Upper**
Heart rate	0.441	0.998	0.993	1.003
Creatinine	0.484	0.839	0.514	1.371
Peak creatine kinase-MB (U/L)	0.512	0.999	0.997	1.001
Peak troponin I	0.149	0.954	0.895	1.017

## Discussion

The main findings of the present study are as follows: 1) Electrocardiographic LVH was observed in ≈ 16% of a 460 consecutive first NSTEMI cohort 2) These patients had higher levels of cardiac troponin but less likely to undergo coronary angiography 3) LVH in ECG was not associated with increased in-hospital complications and mortality.

Though echocardiography is the standard method to diagnose LVH, evaluation of left ventricular hypertrophy by patients’ ECG is easier than other methods and is easily accessible (17, 18). The effect of the ECG LVH on the clinical outcomes of patients with acute coronary syndrome, had been subject of multiple studies with different results. The reported frequency of electrocardiographic LVH in present study is more than the previous studies (8, 11, 12, 13) but approximately in some recent reports (10, 19). Different criteria used in these studies might be the main cause of different frequency. In the present study, LVH patients had significantly higher levels of cardiac enzymes. In the setting of NSTEMI, this may be a new finding as most of previous studies have shown either no difference in troponin or CK-MB level between patients with and without electrocardiographic LVH (12,19) or even lower levels have been reported (8). In contrast, our study results regarding cardiac biomarkers rising resembles one recent study that has been conducted in STEMI patients treated with primary angioplasty and showed higher enzyme rising and infarct size in patients with electrocardiographic LVH [10].One possible mechanism may be related to less cardiac reserve and more myocardial damage in hypertrophied hearts and other mechanism may be due to less capillary density and increased oxygen demand. However, the present study did not measure infarct size with precise imaging techniques such as CMR, so larger studies with accurate myocardial imaging are needed to clarify the exact effect of LVH on infarct size. Similar to previous studies (8, 13), patients with LVH in the present study were less likely to undergo cardiac catheterization but invasive procedure rates were higher than previous studies despite similar age group, as 54% of patients with LVH in this study underwent coronary angiography compared to 43% in the study by Ali et al. and 31% in GUSTO IV ACS study. 

The exact reason of this more conservative approach in the present study is not clear but may related to more advanced age and higher prevalence of comorbidity and frailty in this cohort. Another possible explanation may be related to attribution of ST-segment and T wave changes in ECG to LVH induced ST-T changes and not coronary ischemia by treating physicians and thus selection of conservative approach rather than coronary angiography. Definitely in the present study, we did not measure ST-segment or T wave changes and its prevalence and impact on hospital mortality and this needs further studies. In the present study, no difference was observed in terms of in-hospital complications and death between patients with and without LVH. 

The main possible explanation for no effect of electrocardiographic LVH on in-hospital MACE (major adverse clinical event) may be related to coronary involvement and revascularization procedure in the current study. More than half of patients with LVH had less severe forms of coronary artery stenosis (figure 1) and more than seventy percent of them underwent percutaneous coronary revascularization (figure 2). Hence, better survival is anticipated in this group. Another possible cause may be the higher prevalence of cardioprotective medication use in patients with LVH as 54% and 35% of patients were on beta-blocker and ASA before admission. 

Similar to the present study, Ali et al. concluded that LVH in the ECG had no independent effect on short-term or long-term mortality (13). Moreover, Brown stated that the presence of electrocardiographic LVH at the time of performing PCI did not independently lead to an increase in mortality over a three-year period (14). However, in contrast to the present study, some studies indicated that electrocardiographic LVH was able to predict the death and heart failure (8, 10-12). Westerhout et al. showed that LHV was associated with increased 30-day and one-year death rate; especially in women (8). Nepper-Christensen et al. mentioned that LHV was associated with higher risk of death and heart failure-caused admission in patients treated with primary angioplasty (10). Georgescu et al. stated that LVH criteria in electrography of patients with STEMI who underwent thrombolysis was associated with increased 30-day and one-year mortality (11). 

There is still controversy regarding the role of electrocardiographic LVH in predicting clinical outcomes of patients with ACS which may be due to the following reasons: 1) Difference in sample size; in various studies, hospitalized patients varied from 370 to 8000 people; 2) difference in the use of LVH electrocardiographic criteria. In some studies, Sokolow and in some others, Cornell, or other multivariable criteria were used alone or together, which can be the cause of the difference in the incidence of LVH in patients, and the difference in clinical outcomes of patients. There is some evidence that Sokolow criteria have less sensitivity for diagnosis of LVH than other criteria if echocardiographic data are used as gold standard (20). The main limitations of the present study are: small number of patients with LVH, absence of long-term follow-up, non-randomized, retrospective and single center nature of study as well as lack of detailed echocardiographic data.

Based on the results of the present study, the use of electrocardiographic LVH is not suggested to predict in-hospital outcomes in patients with first NSTEMI. Moreover, considering the different LVH criteria can provide better results. Although, echocardiographic evaluation of patients and long-term follow up may define better role of electrocardiographic LVH in the prediction of patients with first NSTEMI. 

In conclusion,** t**he present study showed that among patients with first NSTEMI, electrocardiographic LVH was not associated with increased in-hospital adverse events.
